# Orthogonality-Constrained CNMF-Based Noise Reduction with Reduced Degradation of Biological Sound

**DOI:** 10.3390/s21237981

**Published:** 2021-11-29

**Authors:** Naoto Murakami, Shota Nakashima, Katsuma Fujimoto, Shoya Makihira, Seiji Nishifuji, Keiko Doi, Xianghong Li, Tsunahiko Hirano, Kazuto Matsunaga

**Affiliations:** 1 Division of Electrical and Electronic Engineering, Graduate School of Sciences and Technology for Innovation, Yamaguchi University, 2-16-1, Tokiwadai, Ube 755-8611, Japan; a052vgu@yamaguchi-u.ac.jp (N.M.); i070fe@yamaguchi-u.ac.jp (K.F.); b048vgv@yamaguchi-u.ac.jp (S.M.); nisifuji@yamaguchi-u.ac.jp (S.N.); 2 Department of Respiratory Medicine and Infectious Disease, Graduate School of Medicine, Yamaguchi University, 1-1-1, Minamikogushi, Ube 755-8505, Japan; decem119@yamaguchi-u.ac.jp (K.D.); lixh@yamaguchi-u.ac.jp (X.L.); tsuna@yamaguchi-u.ac.jp (T.H.); kazmatsu@yamaguchi-u.ac.jp (K.M.)

**Keywords:** noise reduction, biological sound, vascular sound, respiratory sound, wearable device, biomedical signal processing, machine leaning, non-negative matrix factorization

## Abstract

The number of deaths due to cardiovascular and respiratory diseases is increasing annually. Cardiovascular diseases with high mortality rates, such as strokes, are frequently caused by atrial fibrillation without subjective symptoms. Chronic obstructive pulmonary disease is another condition in which early detection is difficult owing to the slow progression of the disease. Hence, a device that enables the early diagnosis of both diseases is necessary. In our previous study, a sensor for monitoring biological sounds such as vascular and respiratory sounds was developed and a noise reduction method based on semi-supervised convolutive non-negative matrix factorization (SCNMF) was proposed for the noisy environments of users. However, SCNMF attenuated part of the biological sound in addition to the noise. Therefore, this paper proposes a novel noise reduction method that achieves less distortion by imposing orthogonality constraints on the SCNMF. The effectiveness of the proposed method was verified experimentally using the biological sounds of 21 subjects. The experimental results showed an average improvement of 1.4 dB in the signal-to-noise ratio and 2.1 dB in the signal-to-distortion ratio over the conventional method. These results demonstrate the capability of the proposed approach to measure biological sounds even in noisy environments.

## 1. Introduction

The number of deaths from cardiovascular diseases such as ischemic heart disease, angina pectoris, and arrhythmia is increasing annually worldwide [1]. Atrial fibrillation is a cardiovascular disease that may cause serious cerebrovascular issues. Atrial fibrillation manifests as an abnormal electrical signal in the atria and causes irregular pulsing and inability to pump sufficient blood [2]. In 2010, 33.5 million patients worldwide were estimated to suffer from this disease. In 2017, this number had increased to 37.6 million [3]. The incidence of this disease has been increasing since the 1990s [4] owing to the population aging. The initial symptoms of atrial fibrillation include abnormalities in the conduction system, such as an irregular pulse. The chronicity of these symptoms can lead to severe cerebral infarction [5,6]. The majority of affected individuals are unaware of the disease because they have no subjective symptoms [7]. Similarly, chronic obstructive pulmonary disease (COPD) is a respiratory disease responsible for an increasing number of deaths. COPD is also a slowly progressing disease and its symptoms originate from limitation of the airflow [8]. This disease is the third leading cause of death worldwide and is expected to become the first in 15 years [9,10]. The initial symptoms of COPD include respiratory abnormalities such as shortness of breath [11].

The initial symptoms of these cardiovascular and respiratory diseases are difficult to detect early by the patient because they are subtle and progress slowly. Currently, the diagnosis of COPD and atrial fibrillation is not automated and requires a visit to a clinician. To improve the efficiency of medical treatment for both diseases, it is necessary to perform repeated objective and accurate tests. This problem can be solved by developing a sensor device that can continuously measure cardiovascular and respiratory conditions. The detection of abnormal signals generated from cardiovascular and respiratory organs by the device will enable clinicians to detect and treat diseases at an early stage. In addition, the collection and analysis of measurement data can be applied as a diagnostic aid for physicians or for automated diagnosis. Several biological measurement devices have already been commercialized, such as bedside monitors and electronic stethoscopes used in hospitals [12,13]. The bedside monitor displays multiple vital signs, such as the electrocardiogram, respiratory information, and body temperature of the patient. The electronic stethoscope can record vascular and respiratory sounds even in noisy environments. However, these devices are not always available for measurement and it is difficult for people without medical knowledge to evaluate the measured data. Hence, simultaneous evaluation systems for vascular and respiratory sounds are being researched worldwide. In prior research, we developed a biological sound sensor system that could measure vascular and respiratory sounds simultaneously [14]. This system simultaneously measures these sounds from the trachea and arteries through contact with the human body, and extracts these sounds through biomedical signal processing. This development enabled patients to easily measure their own vascular and respiratory sounds. However, the biological sound sensor was negatively affected by noise from the user’s surroundings.

Over the years, researchers have proposed methods to reduce noise in measured sounds. Examples of noise reduction methods include the application of Wiener filters, spectral subtraction, and empirical mode decomposition (EMD) [15,16,17,18,19]. These reduction effects are related to the number of microphones and the computational complexity. From the viewpoint of miniaturization in wearable devices, a noise reduction method that functions with only a single microphone is desirable. A noise reduction method for monaural sources was formulated based on non-negative matrix factorization (NMF). It has been reported that the noise reduction performance of NMF is superior to those of methods using EMD and other methods described above [20]. NMF decomposes a non-negative matrix into two matrices; it has been widely applied in various fields such as image processing, text analysis, and speech processing [21,22,23]. In our previous study, we proposed a noise reduction method based on semi-supervised convolutive non-negative matrix factorization (SCNMF), which is an extension of NMF [24]. Vascular and respiratory sounds have a time dependence on the frequency; SCNMF was developed for the analysis of these sounds. Nevertheless, this method may misclassify some parts of biological sounds as noise and consequently distort biological sounds. In this paper, we propose a novel NMF extension model, orthogonality-constrained convolutive NMF (OCNMF), which imposes a similarity constraint between noise and biological sounds on the SCNMF. The OCNMF-based noise reduction method can prevent the distortion of biological sounds due to misclassification. The proposed noise reduction can contribute greatly to health care innovations in biomedical signal processing, such as heartbeat analysis and blood pressure prediction [25,26]. The effectiveness of OCNMF was verified experimentally by using biological sounds acquired from 21 subjects.

This paper is organized as follows. Section 2 explains the vascular and respiratory sounds and the structure and principles of biological sound sensors. Section 3 discusses the related research and explains the proposed OCNMF. Section 4 describes the experimental setup and verification process. Finally, Section 5 concludes the paper.

## 2. Biological Sound Sensor

Biological sounds such as vascular, respiratory, and swallowing sounds are generated in the body through physiological functions. Biological sounds are one of the most important sources of information about a person’s health status. Hence, physicians perform initial diagnosis through auscultation. We designed a sensor to measure vascular and respiratory sounds for diagnosing cardiac and pulmonary diseases. In the following subsections, we explain vascular and respiratory sounds and the structure and principle of the biological sound sensor used in this study.

### 2.1. Vascular Sound

Vascular sound is generated by the opening and closing of the heart valves due to beating. This sound is divided into four sounds: S1, S2, S3, and S4. S1 is generated by the closing the mitral and tricuspid valves and S2 is generated by the closing of the aortic and pulmonary valves. S3 and S4 have low-frequency components and are identified by their very low amplitude. Therefore, it is difficult to hear S3 and S4 [27]. Vascular sounds have a main frequency range of 75–200 Hz [28].

### 2.2. Respiratory Sound

Respiratory sounds are generated by the flow of air through the airway during exhalation and inhalation [29]. This sound has a frequency range of 200–2000 Hz for both exhalation and inhalation. The high-frequency components are attenuated by the propagation medium in the body, such as bone and soft tissue. For example, the respiratory sound generated by the chest has a frequency range of approximately 200–600 Hz [30].

### 2.3. Biological Sound Sensor

**Figure 1:**
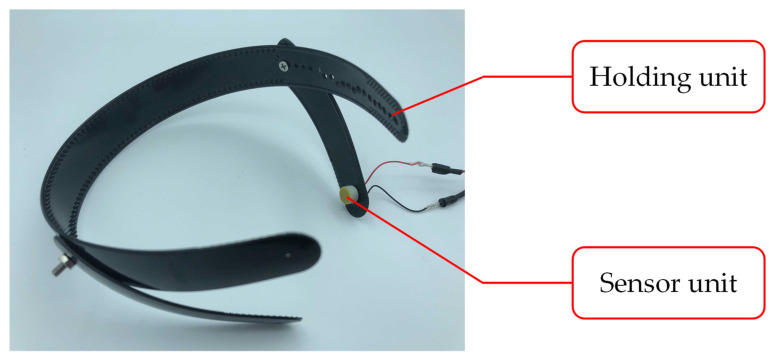
Biological sound sensor.

**Figure 2:**
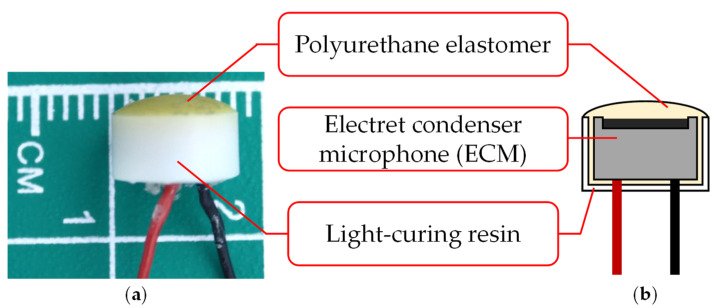
Sensor unit: (**a**) photograph; (**b**) cross section.

**Figure 3:**
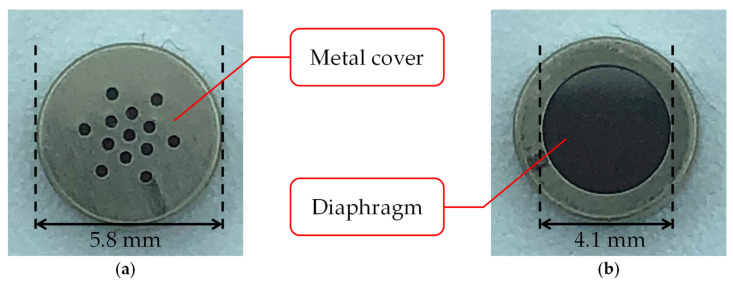
Electret condenser microphone (ECM): (**a**) before exposure; (**b**) after exposure.

The biological sound sensor shown in Figure 1 is a wearable sensor that can measure vascular and respiratory sounds [14]. The sensor used in this study consists of two parts: a holding unit and a sensor unit. Figure 2 shows the photograph and cross-sectional illustration of the sensor unit. The holding unit is made of an elastoplastic material. The unit is designed to keep the sensor unit in contact with the human body. The sensor unit consists of an electret condenser microphone (ECM), a contact part, and a case. The ECM EM-258 (Primo Co., Ltd., Tokyo, Japan) was designed with its diaphragm exposed to accurately measure biological sounds. Figure 3 shows the ECM before and after exposure. The ECM is housed in a light-curing resin case, and its exposed diaphragm is covered with a polyurethane elastomer with a hardness of 15 HITOHADA gel (Exseal Co., Ltd., Gifu, Japan).

The ECM is a condenser microphone with an electret. The condenser microphone consists of a capacitor with two electrodes and a diaphragm. The distance between the two electrodes is lengthened or shortened by vibrations of the input sound wave. This mechanism converts the sound vibrations into voltage changes, which enables the condenser microphone to receive sound as an electrical signal. The condenser microphones require a high voltage, known as a phantom source, for charging. In contrast, the ECM can be used at low voltages because it has an electret element that can be charged semi-permanently [31]. The ECM is suitable for wearable devices because of its small size and non-requirement of additional power supply. Signal processing is necessary to compensate for the lower sound quality of the ECM when compared with condenser microphones.

The polyurethane elastomer is used to accurately measure biological sounds with the ECM. The biological sounds are attenuated at the interface between the skin and the sensor because of the natural law that sound waves propagating in a medium with different acoustic properties will be reflected. Their acoustic impedances are matched by passing through a polyurethane elastomer, which has acoustic properties similar to those of the skin.

**Figure 4:**
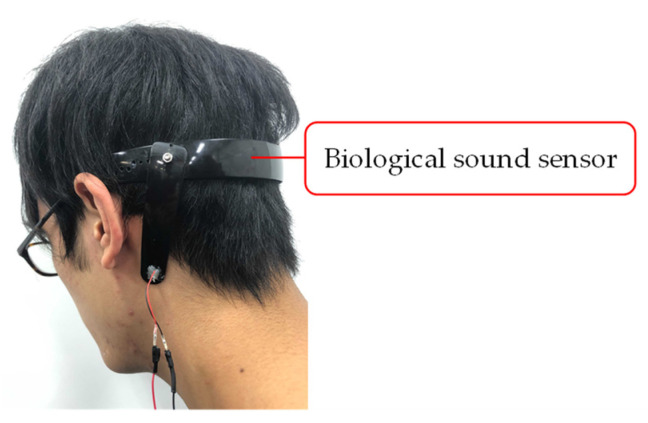
Photograph of a subject wearing the biological sound sensor.

We decided to measure the biological sound around the mastoid process of the subjects. The mastoid process is a cone-shaped bony prominence located in the posterior inferior portion of the temporal bone [32]. This position enables simultaneous measurement of vascular and respiratory sounds because of its proximity to blood vessels and the trachea. Furthermore, it alleviates the discomfort felt by the user when wearing the sensor. Figure 4 shows a photograph of a subject wearing the biological sound sensor.

## 3. Noise Reduction Methods

### 3.1. Related Research

In the field of audio signal processing, noise reduction methods have been designed with a focus on time and frequency properties. In the following subsections, we introduce non-negative matrix factorization (NMF), a monaural noise reduction method that has been studied extensively in recent years, and its extended models.

#### 3.1.1. NMF and Semi-Supervised NMF

NMF is a method for decomposing non-negative data into additive components [21,22,23]. Based on previous studies, additivity is assumed valid in an amplitude spectrogram, which is an absolute value of the audio signal processed by short-time Fourier transform (STFT). Given a non-negative matrix ***Y*** ∈ ${\mathbb{R}}_{+}^{F\times T}$, NMF approximates it by the product ***Y*** ≅ ***HU*** of a basis matrix ***H*** ∈ ${\mathbb{R}}_{+}^{F\times R}$ and an activation matrix ***U*** ∈ ${\mathbb{R}}_{+}^{R\times T}$, where the input ***Y*** is the amplitude spectrogram of the audio signal, *F* is the number of frequency bins, *T* is the number of frames, and *R* is the number of bases of the NMF. ***H*** contains the spectral patterns found in the original spectrogram and ***U*** contains the time variation of the amplitude of each spectral pattern. The computational complexity and decomposition accuracy of NMF depend on an objective function that represents the norm between the target matrix and the output.

The objective functions of the NMF have been proposed by many researchers. In this study, we focus on *β*-divergence, which was devised in previous studies [33,34]. The *β*-divergence is expressed in Equation (1) using the variables *β*, *x*, and *y*. In Equation (1), $D_{\beta }\left(y|x\right)$ is the pseudodistance of *y* to *x*. This is the Itakura–Saito (IS) distance when *β* = 0, the Kullback–Leibler (KL) distance when *β* = 1, and the Euclidean distance when *β* = 2.

(1) $$D_{\beta }\left(y|x\right)=\frac{y^{\beta }}{\beta \left(\beta -1\right)}+\frac{x^{\beta }}{\beta }\;-\;\frac{{\mathrm{yx}}^{\beta -1}}{\beta -1}$$

The objective function of the NMF with *β*-divergence as the norm is expressed in Equation (2).

(2) $$E_{\beta }\left(\theta \right){=D}_{\beta }\left(Y|\mathrm{HU}\right),$$

where *θ* is the parameter to be optimized. NMF estimates ***H*** and ***U*** such that $E_{\beta }\left(\theta \right)$ is minimized. It is not possible to solve this optimization problem analytically. However, the solution can be obtained indirectly by iteratively minimizing the auxiliary function, which is the upper bound of the objective function [35]. The multiplicative update rules for the objective function with *β*-divergence are expressed as follows:

(3) $$H\;\leftarrow \;H\;⨀{\left(\frac{{\left(\mathrm{HU}\right)}^{\beta -2}{\mathrm{YU}}^{T}}{{\left(\mathrm{HU}\right)}^{\beta -1}U^{T}}\right)}^{\varphi (\beta )},$$

(4) $$U\;\leftarrow \;U\;⨀{\left(\frac{H^{T}{\left(\mathrm{HU}\right)}^{\beta -2}Y}{H^{T}{\left(\mathrm{HU}\right)}^{\beta -1}}\right)}^{\varphi (\beta )},$$

where the operator ⨀ represents the adamantine product, and the operator (·)^*T*^ represents the transpose operation. φ(β) is defined using the following equation:

(5) $$\varphi \left(\beta \right)=\{\begin{array}{ll} 1/\left(2-\beta \right) & \left(\beta <1\right)\\ 1 & \left(1\leq \beta \leq 2\right)\\ 1/\left(\beta -1\right) & \left(2<\beta \right) \end{array}.$$

It is difficult to interpret the meaning of the spectral patterns represented by each basis because NMF is an unsupervised learning process. In addition, a single basis may contain extensive signal information. Thus, the extraction of the target signal using NMF is very problematic. 

Target signal extraction methods have been devised using the spectral patterns of the signal as supervised information [36,37,38]. Semi-supervised NMF (SNMF) is an extension of NMF to supervised clustering. SNMF consists of two phases: pre-training and noise analysis. In pre-training, the amplitude spectrogram ***Y****_target_* ∈ ${\mathbb{R}}_{+}^{F\times T}$ of the target signal sound without noise is decomposed to the product of ***H*** ∈ ${\mathbb{R}}_{+}^{F\times R}$ and ***Q*** ∈ ${\mathbb{R}}_{+}^{R\times T}$ through NMF. The basis matrix ***H*** is incorporated into the NMF as supervised information for the target signal. In noise analysis, ***Q*** is not necessary because it represents the activity level of the pre-trained sound. SNMF decomposes the amplitude spectrogram ***Y*** ∈ ${\mathbb{R}}_{+}^{F\times T}$ of the unknown mixed signal, as shown in the following equation:

(6) Y ≅  HU+FG,

where ***F*** ∈ ${\mathbb{R}}_{+}^{F\times J}$ is the basis matrix that contains information other than the signal to be extracted in the noisy input signal, ***G*** ∈ ${\mathbb{R}}_{+}^{J\times T}$ is the activation matrix corresponding to ***F***, and *J* is the number of bases in the basis matrix ***F***. ***H*** is the basis matrix of the extraction target signal obtained through pre-training. Note that ***H*** is not updated during the decomposition of Equation (6) because ***H*** contains the spectral pattern of the extraction target signal. The extraction target signal can be separated from the other signals by decomposing the input sound into additive components, as described above. The objective function $I_{\beta }\left(y|x\right)$ of SNMF with *β*-divergence is defined as follows:

(7) $$I_{\beta }\left(\theta \right){=D}_{\beta }\left(Y|\mathrm{HU}+\mathrm{FG}\right).$$

The multiplicative update rules that minimize the objective function of SNMF using the auxiliary function as well as Equations (3) and (4) can be derived as follows:

(8) $$F_{\;}\leftarrow \;F\;⨀{\left(\frac{{\left(\mathrm{HU}+\mathrm{FG}\right)}^{\beta -2}{\mathrm{YG}}^{T}}{{\left(\mathrm{HU}+\mathrm{FG}\right)}^{\beta -1}G^{T}}\right)}^{\varphi (\beta )},$$

(9) $$G_{\;}\leftarrow \;G\;⨀{\left(\frac{F^{T}{\left(\mathrm{HU}+\mathrm{FG}\right)}^{\beta -2}Y}{F^{T}{\left(\mathrm{HU}+\mathrm{FG}\right)}^{\beta -1}}\right)}^{\varphi (\beta )},$$

(10) $$U_{\;}\leftarrow \;U\;⨀{\left(\frac{H^{T}{\left(\mathrm{HU}+\mathrm{FG}\right)}^{\beta -2}Y}{H^{T}{\left(\mathrm{HU}+\mathrm{FG}\right)}^{\beta -1}}\right)}^{\varphi (\beta )}.$$

SNMF is utilized as a noise reduction method by reconstructing the sound from the information ***HU*** of the target signal in the mixed signal obtained in this manner. 

SNMF is based on the regular NMF, which assumes that the spectral pattern of the audio signal is time-invariant. This assumption has been reported to deteriorate the extraction accuracy for signals with time-varying amplitude spectra, such as voices [39]. In the next subsection, we describe the convolutive NMF for time-varying signal analysis.

#### 3.1.2. Convolutive NMF and Semi-Supervised Convolutive NMF

Convolutive NMF (CNMF) is an extended NMF capable of analyzing audio signals with a time-varying amplitude spectrum [40]. Convolution is an operation used to estimate the relationship between the neighboring spectra on the time axis. CNMF decomposes the amplitude spectrogram ***Y*** of the audio signal as follows:

(11) Y ≅ ∑τ=0K−1HτU→τ,

where $H_{\tau }$∈${\mathbb{R}}_{+}^{F\times R}$ represents the basis matrix and *K* represents the number of time frames to be convolved. Note that the operator U→τ shifts the matrix column to the right by τ and sets the elements of the shifted column to zero from outside the matrix. In contrast, the operator U←τ shifts the matrix column to the left by τ and sets the elements of the shifted column to zero from outside the matrix. As shown in Equation (11), CNMF decomposes the amplitude spectrogram of the audio signal into a shared activation matrix and a set of time-frame-shifted basis matrices. From this calculation, CNMF can estimate the time variation and relationship between the neighboring spectra.

**Figure 5:**
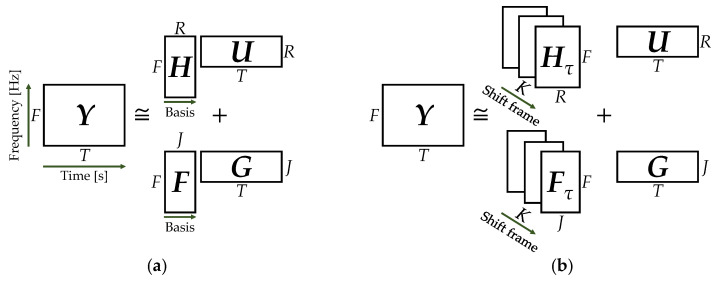
Conceptual diagram of semi-supervised non-negative matrix factorization (SNMF) and semi-supervised convolutive NMF (SCNMF): (**a**) SNMF; (**b**) SCNMF.

Semi-supervised CNMF (SCNMF) is an extended model of SNMF that utilizes CNMF for pre-training and noise analysis. Figure 5 shows a conceptual diagram of SNMF and SCNMF. It is noteworthy that the spectral pattern of respiratory sounds changes from the beginning to the end of the respiratory cycle. We have already reported that SCNMF has a high noise reduction effect in respiratory sounds with a time-varying frequency distribution [24].

The objective function $L_{\beta }\left(\theta \right)$ of SCNMF and its multiplicative update rules derived as followed in Equations (8)–(10) are given below. Note that the estimated result of the SCNMF, ***Z***, is defined in Equation (12).

(12) Z =∑τ=0K−1HτU→τ+∑τ=0K−1FτG→τ

(13) $$L_{\beta }\left(\theta \right){=D}_{\beta }\left(Y|Z\right),$$

(14) Fτ ← Fτ ⨀(Z β−2Y U→TτZ β−1G→Tτ)φ(β),

(15) G ← G ⨀(FτTZ←τY←τβ−2FτTZ←τ β−1)φ(β),

(16) U ← U ⨀(HτTZ←τY←τβ−2HτTZ←τ β−1)φ(β).

Through these multiplicative update rules, information other than the target signal in the input spectrogram is contained in the basis matrix ***F***. However, NMF and SCNMF have the risk of incorrectly storing the target signal in ***F***, because NMF originally has no uniqueness and has high dependency on the initial value in the solution. In the next subsection, we describe the details of an extended model of the SNMF, called the orthogonality-constrained NMF, which solves this problem.

#### 3.1.3. Orthogonality-Constrained NMF

A method has been devised to improve the separation accuracy of the target signal by imposing constraints on the multiplicative update rules of the SNMF. Orthogonality-constrained NMF (ONMF) imposes a constraint that maximizes the cosine distance between the pre-training basis matrix and the other basis matrices [41]. By orthogonalizing the basis vectors of the pre-training basis matrix ***H*** with those of the other basis matrices ***F***, they become uncorrelated. ONMF imposes the following constraints on the product of ***F*** and ***H***.

(17) $$\underset{F}{\min}{\left(\mathrm{HF}\right)}^{2}$$

With the imposition of this constraint, the objective function converges by normalizing each column of the basis matrix. The update rule for each element $F_{f,j}$ of the basis matrix ***F*** other than the target signal can be obtained by solving the optimization problem of the auxiliary function, which is the upper bound of the objective function. The update rule is as follows:

(18) $$F_{f,j}\leftarrow {\;F}_{f,j}\;{\left(\frac{\sum_{t}{\left(\sum_{r}H_{f,r}U_{r,t}+\sum_{j}F_{f,j}G_{j,t}\right)}^{\beta -2}Y_{f,t}G_{j,t}}{\sum_{t}{\left(\sum_{r}H_{f,r}U_{r,t}+\sum_{j}F_{f,j}G_{j,t}\right)}^{\beta -1}G_{j,t}{+\mu F}_{f,j}\sum_{r}H_{f,r}^{2}}\right)}^{\varphi (\beta )},$$

where $H_{f,r}$ denotes each element of the basis matrix ***H***, $U_{r,t}$ belongs to the activation matrix ***U***, $G_{j,t}$ belongs to the activation matrix ***G***, and $Y_{f,t}$ is the input amplitude spectrogram ***Y***. The parameter μ is the weight variable of the orthogonality constraint. When the parameter μ = 0, the ONMF process is the same as that of the regular SNMF. In the next subsection, a novel noise reduction method based on these NMF models is proposed to prevent distortion of the respiratory sound signal information.

### 3.2. Proposed Method

**Figure 6:**
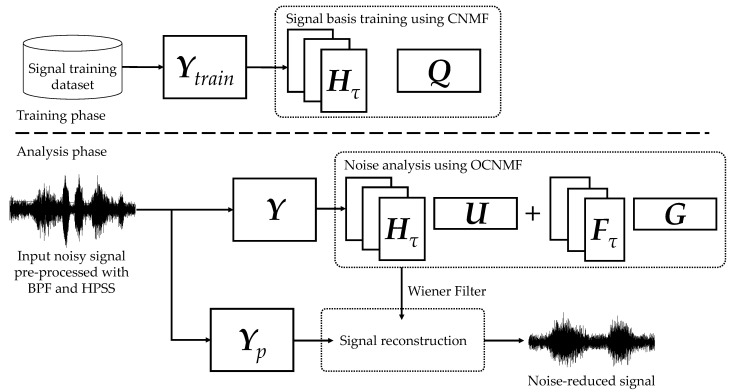
Proposed noise reduction framework based on orthogonality-constrained CNMF.

We propose an orthogonality-constrained convolutive NMF (OCNMF) framework for noise reduction, as shown in Figure 6. First, the biological sound measured by the sensor is preprocessed using a band-pass filter (BPF) and harmonic percussion sound separation (HPSS), as described below, to separate the sound into vascular and respiratory sounds. The purpose of this preprocessing is to independently learn vascular and respiratory sounds, which have different characteristics, as described above. We used BPF and HPSS, which are fast processing methods, because the focus of this study is to improve the efficiency of healthcare. Second, the separated respiratory sounds are converted into an amplitude spectrogram by using STFT for OCNMF analysis. OCNMF consists of pre-training and noise analysis phases as well as SNMF. In pre-training, OCNMF learns a dataset, $Y_{\mathrm{train}}$ ∈ ${\mathbb{R}}_{+}^{F\times T_{\mathrm{train}}},$ which consists only of signal information because it requires no information of unknown noise. In the noise analysis, OCNMF decomposes the preprocessed as well as the pre-training input amplitude spectrogram Y. The mask M shown in Equation (19) is generated from the matrices obtained through OCNMF and post-processed with a Wiener filter multiplied by Y to reconstruct the amplitude spectrogram of the denoising result.

(19) M =(∑τ=0K−1HτU→τ)2(∑τ=0K−1HτU→τ)2+(∑τ=0K−1FτG→τ)2

Finally, the product of the denoised amplitude spectrogram and the phase of the input signal $Y_{p}$ are processed using inverse STFT (ISTFT) to obtain the denoised signal. In the following subsections, we explain the preprocessing and details of the proposed OCNMF. 

#### 3.2.1. Preprocessing with BPF and HPSS

To pre-determine the number of bases other than the target signal, the SNMF should narrow down the types of sounds in the preprocessing. Frequency filters designed to match the frequency distribution of vascular and respiratory sounds can extract the respective sounds [14]. The specifications of the BPF designed in this study are listed in Table 1. Our system is intended for practical use in telemedicine and is expected to demonstrate real-time performance. Hence, we designed an infinite impulse response (IIR) filter with low computational complexity. A Butterworth filter was selected because the signal distortion caused by the passband ripple is more detrimental to OSCNMF than the slower out-of-band attenuation. The order was set to 12 to sufficiently attenuate the out-of-band frequency components. Nevertheless, this BPF process is not sufficient to completely extract only the respiratory sounds. The reasons for this are the wide range of secondary frequency components of vascular sounds and the pressure of these sounds, which is approximately 20 dB higher than that of the respiratory sounds.

In this paragraph, we describe the characteristics of vascular and respiratory sounds and explain the process of removing residual vascular noise from respiratory sounds. As mentioned in the previous paragraph, vascular sounds are short in the time axis and wide in the frequency axis. In contrast, respiratory sounds are longer in the time axis and narrower in the frequency axis. HPSS can be used to separate such sources with temporal and frequency differences [42]. Vascular sounds have percussive characteristics and respiratory sounds have harmonic characteristics. HPSS first applies a one-dimensional median filter with a predefined filter width to each column and row of the amplitude spectrogram of the mixed signal. While setting this filter width in the HPSS, it is necessary to consider the time width of the harmonic and percussive sounds to be extracted. The following conditional equation is imposed on the filter width $m_{t}$ in the time axis direction for harmonic extraction, which depends on the time width $p_{t}$ of the percussive sound and time width $h_{t}$ of the harmonic sound.

(20) $${2p}_{t}{\;<\;m}_{t}{\;<\;h}_{t}$$

In this study, the filter width was set to a time frame equivalent to 0.61 s based on the time length of the respiratory and vascular sounds. A mask was calculated from the respiratory sound enhanced by the median filter, as in Equation (19), and multiplied by the original mixture signal to obtain the separation result.

The HPSS processes any signal with a predefined filter width. Hence, it has the risk of causing musical noise by over-reducing when unexpected noise characteristics are mixed in [43]. Our proposed OCNMF is expected to recover from the degradation caused by non-adaptive HPSS because it reduces the noise of the input based on the supervised signal.

#### 3.2.2. Noise Reduction with OCNMF

As mentioned in the previous subsection, CNMF is effective in analyzing signals with time-varying frequency patterns, such as biological sounds. Based on Equation (11), the multiplicative update rules for the basis matrix $H_{\tau }$ and activation matrix ***Q*** of the target signal information are given as follows:

(21) Hτ ← Hτ ⨀(Zβ−2YtrainQ→Tτ Z β−1Q→Tτ)φ(β),

(22) Q ← Q ⨀(HτTZ←τYtrain←τβ−2HτTZ←τ β−1)φ(β),

where ***Z*** is the estimated result of CNMF. The procedure for pre-training using CNMF is outlined in Algorithm 1. $i_{\mathrm{CNMF}}$ represents the number of update rule iterations required for solution convergence. The number of iterations, $i_{\mathrm{CNMF}}$, depends on the size of the input matrix and the number of shift frames *K*. A previous study reported that it converged at approximately 100 [39]. The *β*-divergence should be determined based on the NMF model characteristics and the target sound generation process. NMF with the IS, KL, and Euclidean distances as norms is equivalent to the maximum likelihood estimation assuming exponential, Poisson, and Gaussian distributions for the generation process [44].
**Algorithm 1.** Signal basis training using CNMF**Input:** Spectrogram of signal dataset $Y_{\mathrm{train}}$, priori basis number *R*, shift length of CNMF *K*, type of *β*-divergence *β*   number of iterations in CNMF $i_{\mathrm{CNMF}}$
**Output:** The basis matrix of signal $H_{\tau }$
1:  Initialize $H_{\tau }$ and Q with random non-negative values2:  Normalize columns of $H_{\tau }$
3:  **for** *i* = 1, ⋯, $i_{\mathrm{CNMF}}$ **do**4:   **for**
τ = 0, ⋯, *K* − 1 **do**5:    Compute ***Z***6:    Update $H_{\tau }$ and Q using Equations (21) and (22)7:    Normalize columns of $H_{\tau }$
8:   **end for**9:  **end for**

**Figure 7:**
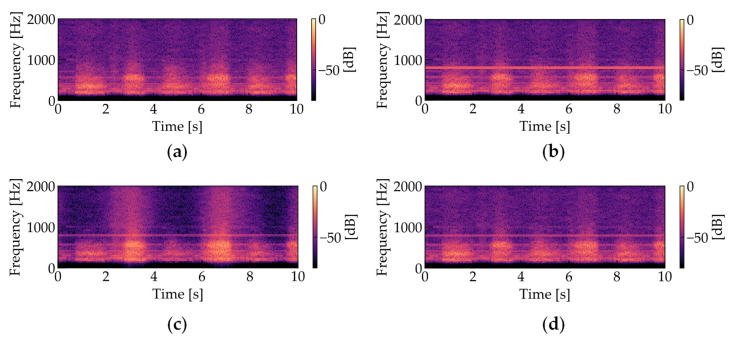
Amplitude spectrograms of respiratory sounds (The parameters were set as follows: sampling frequency = 44.1 kHz, window length in STFT = 1024 points, window length in STFT = 512 points): (**a**) noiseless; (**b**) noise-added; (**c**) processed with SCNMF (*R* = 30, *J* = 15, *K* = 10, $i_{\mathrm{CNMF}}$ = 200, $i_{\mathrm{SCNMF}}$ = 200, *β* = 2); (**d**) processed with the proposed OCNMF (*R* = 30, *J* = 15, *K* = 10, $i_{\mathrm{CNMF}}$ = 200, $i_{\mathrm{OCNMF}}$ = 200, *β* = 2, μ = 1.0 × 10^6^).

OCNMF imposes a constraint similar to Equation (17) on the product of the basis matrix $H_{\tau }$ of the target signal information and the basis matrix $F_{\tau }$ of the noise. Based on the constraints, the update rule for each element $F_{\tau ,f,j}$ of $F_{\tau }$ in OCNMF is given as follows:

(23) $$F_{\tau ,f,j}\leftarrow {\;F}_{\tau ,f,j}\;{\left(\frac{\sum_{t}Z_{f,t}{}^{\beta -2}Y_{f,t}G_{j,t}}{\sum_{t}Z_{f,t}{}^{\beta -1}G_{j,t}{+\mu F}_{\tau ,f,j}\sum_{r}H_{\tau ,f,r}^{2}}\right)}^{\varphi (\beta )},$$

where ***Z*** is the estimated result of the OCNMF denoted by Equation (12). Note that the convergence of OCNMF is conditional on the normalization of the basis vectors, as is the case with ONMF. Noise analysis using OCNMF and noise reduction through masking are performed as shown in Algorithm 2. Figure 7 shows the amplitude spectrograms of the four types of respiratory sounds (Figure 7a: noiseless, Figure 7b: noise added, Figure 7c: processed using SCNMF, Figure 7d: processed using the proposed OCNMF method). Based on preliminary experiments investigating the time length of changes in the frequency pattern of the respiratory sound, the number of shift frames *K* is set to be equivalent to 0.3 s. To scale the two terms in the denominator of the right side of Equation (23), the weight parameter μ of the orthogonality constraint was set to 1.0 × 10^6^. As shown in Figure 7c,d, our proposed OCNMF has less respiratory sound distortion than the SCNMF.
**Algorithm 2.** Noise analysis and reduction using OCNMF**Input:** Spectrogram of an input signal Y, priori basis number *R*, an undesired basis number *J*, type of *β*-divergence *β*,   number of iterations in OCNMF $i_{\mathrm{OCNMF}}$, shift length of CNMF *K*, signal basis matrix $H_{\tau }$,   weight parameter μ, phase matrix of the input signal $Y_{p}$
**Output:** Noise reduced signal $Y_{\mathrm{cl}}$
1:  Initialize $F_{\tau }$, ***U*,** and ***G*** with random non-negative values2:  Normalize columns of $F_{\tau }$
3:  **for** *i* = 1, ⋯, $i_{\mathrm{OCNMF}}$ **do**4:   **for**
τ = 0, ⋯, *R* − 1 **do**5:    Compute ***Z***6:    Update $F_{\tau }$, ***U***, and ***G*** using Equations (15), (16) and (23)7:    Normalize columns of $F_{\tau }$
8:   **end for**9:  **end for**10:  Compute ***M*** using Equation (19) and $Y_{\mathrm{cl}}$ = Y ⨀ ***M***11:  **ISTFT** ($Y_{\mathrm{cl}}$ ⨀ $Y_{p}$)

## 4. Experimental Verification

### 4.1. Setup

An experiment was conducted to verify the effectiveness of the noise reduction based on our proposed OCNMF. Sounds that were measured in noiseless environments were used as the pre-training data, and sounds to which noise was added on the computer were used as the test data. The method of separating the pre-training data and test data is explained in the next subsection. As shown in Figure 3, the biological sound sensor was attached to the area around the mastoid process of a subject in a seated resting state. The sensor was connected directly to the PC with a phone connector for power supply and recording control. Biological sounds were measured for 10 s in 21 subjects who consented to participate in this study. The health status of the subjects is shown in Table 2. The experimental design was approved by the Institutional Review Board of Yamaguchi University (Approval number: H2021-031). The respiratory rate was arbitrarily set by the subjects. The sampling frequency and bit depth of the measurement were set to 44.1 kHz and 16 bits, respectively, which assured the same sound quality as that from a CD. This was because the future goal of our research is to realize telemedicine and automated auscultation. Two types of noises were added: a sine wave of 800 Hz and a male voice with frequency range similar to that of the respiratory sound. The sine wave noise simulated stationary noise, while the male voice speaking Japanese sentences simulated non-stationary noise. To quantitatively evaluate the noise reduction results, the noises were adjusted to have the same maximum amplitude as the respiratory sounds. Table 3 lists the parameters of STFT and OCNMF. To reduce respiratory sound distortion, the cost function was set to the Euclidean distance (*β* = 2), which evaluates both positive and negative norms equally. The effectiveness of the proposed OCNMF method was evaluated by comparing the signal-to-noise ratio (SNR) and signal-to-distortion ratio (SDR) with the results of the conventional SCNMF method. SNR is commonly used to evaluate the sound quality after noise reduction and SDR is commonly used to evaluate the accuracy of noise separation [45,46,47]. The details of the evaluation method are described in the following subsection.

### 4.2. Evaluation Methodology

The measured biological sound signals were divided into one unit of test data and the remaining as pre-training data based on the leave-one-out method [48]. The leave-one-out method is a type of cross-validation that is used in cases where a large amount of data cannot be prepared. In this experiment, it was difficult to collect a large number of biological sounds because we used our own sensors. Hence, the leave-one-out method was adopted.

To verify the effectiveness of noise reduction using both methods, noise was added to the test data on the computer. The test data were adjusted such that 0 to 5 s consisted of biological sound only, 5 to 10 s consisted of the biological sound and noise, and 10 to 15 s consisted of noise only, for a total of 15 s. The SNR was calculated from the time signals of the first 5 s and the last 5 s, and the SDR was calculated from the time signal of the first 10 s. The SNR was defined using the time signal *s*(*t*) of only the biological sound and the time signal *n*(*t*) of only noise, as shown in the following equation:

(24) $${\mathrm{SNR}}_{\;}{=10\log}_{10}\frac{\max\left(\left|s\left(t\right)\right|\right)}{\max\left(\left|n\left(t\right)\right|\right)}\;\;\;[\mathrm{dB}],$$

where the operator |·| denotes the absolute value. As shown in Equation (24), the SNR can evaluate sound quality using the amplitude ratio of the signal to noise in mixed sounds. Note that the higher the SNR, the higher the sound quality. The SNR of the test data before noise reduction in this experiment was set to 0 dB and the difference between this value and the SNR after noise reduction quantitatively evaluates the effectiveness of noise reduction. The SDR was calculated from the time signals before and after the noise reduction process; it indicates the level of signal distortion caused by the noise reduction process. The defining equation of SDR based on the time signal before the noise reduction process *b*(*t*) and the time signal after the process *a*(*t*) is as follows:

(25) $${\mathrm{SDR}}_{\;}{=10\log}_{10}\frac{\sum_{t}^{S\;-\;1}b^{2}\left(t\right)}{\sum_{t}^{S-1}{\left\{b\left(t\right)-\;\lambda a\left(t\right)\right\}}^{2}}\;[\mathrm{dB}],$$

where *S* represents the number of samples for the time signal and *S* = 441 × 10^3^ (equivalent to 10 s). *λ* is a parameter that adjusts the volume before and after noise reduction and is defined as the following equation:

(26) $$\lambda_{\;}=\sqrt{\frac{\sum_{t}^{S-1}b^{2}\left(t\right)}{\sum_{t}^{S\;-\;1}a^{2}\left(t\right)}}.$$

### 4.3. Results and Discussion

**Figure 8:**
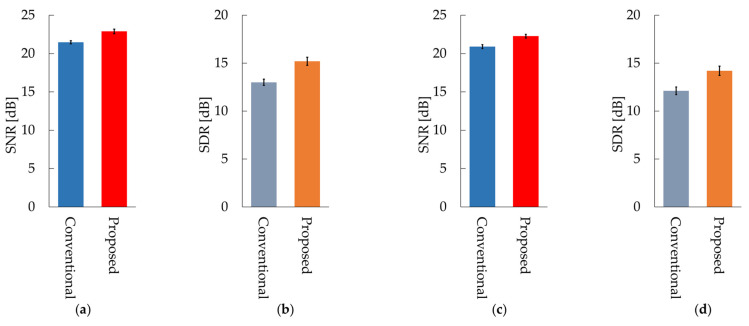
Results of the conventional method SCNMF and proposed method OCNMF: (**a**) signal-to-noise ratio (SNR) in 800 Hz sine wave noise; (**b**) signal-to-distortion ratio (SDR) in 800 Hz sine wave noise; (**c**) SNR in male voice noise; (**d**) SDR in male voice noise.

Figure 8 shows the average values of the SNR and SDR for all subjects for the conventional and proposed methods. Table A1 in Appendix A shows the SNR and SDR for each subject in the case of sine wave noise and Table A2 shows the SNR and SDR for each subject in the case of male voice noise. The SNR and SDR of our proposed OCNMF were significantly higher than those of the SCNMF for both noises (*p* < 0.001).

The following is a discussion of the experimental results. In the case of sine wave noise, the SNR of the proposed OCNMF method exceeded that of the conventional SCNMF method for almost all subjects. The reason for this result is that the noise basis matrix contains a sine wave with a different frequency pattern from the signal basis vector owing to the orthogonality constraint imposed on the noise basis matrix. This result suggests that OCNMF is effective in reducing stationary noise. The SDR of OCNMF exceeded that of SCNMF for all subjects, with a minimum difference of 0.2 dB and a maximum difference of 5.5 dB. This was because OCNMF was able to solve the problem of SCNMF, wherein the respiratory sound in the noise mixture signal was incorrectly contained in the noise basis matrix. This finding indicates that the orthogonality constraint is useful for reducing respiratory sound distortion. Likewise, the SNR of OCNMF exceeded the SNR of SCNMF for almost all subjects in the case of male voice noise. A previous study reported that an SNR of approximately 20 dB is sufficient for calculating the respiratory rate from respiratory sounds [24]. The SNRs of the experimental results were mostly above 20 dB, suggesting that our proposed method is sufficiently reliable for biological measurements. However, the SNR of subject L was 1.34 dB lower. The reason for this result could be that the frequency pattern of the added noise was similar to that of subject L’s respiratory sound. The order of the parameter *μ* in the signal and noise similarity constraint was set on the basis of the number of elements in the input spectrogram in the experiments. For commercialization as a wearable healthcare device, the parameter *μ* should be carefully set according to the quality of the pre-training data and applications such as the detection of abnormal respiratory sounds. For example, in the case of pre-training with normal respiratory sounds that are relatively easy to collect and utilize in the abnormal respiratory sound detection system, a very high value of the parameter *μ* will cause the abnormal respiratory sounds to be removed as noise. The results of SDR using OCNMF in male voice noise exceeded those of the conventional method in almost all subjects. These results support the premise of our proposed method that orthogonality constraints can emphasize respiratory sounds without distortion.

Incidentally, the experimental results should be interpreted with caution, as they may be influenced by the similarity of the subjects’ respiratory sounds. Most of the subjects in this experiment were patients with respiratory diseases, such as asthma or COPD. It has been reported that the spectral distribution of respiratory sounds differs for each individual [49,50]; thus, it is necessary to further verify whether the spectral distribution added to the signal basis matrix by pre-training is a generalized respiratory sound. Future studies are required to analyze the effects of individual differences in respiratory sounds by examining a large sample size, including people with other diseases.

## 5. Conclusions

The aging population has resulted in increasing mortality due to cardiovascular and respiratory diseases. To detect the early symptoms of these diseases, a device that can constantly evaluate biological sounds in a user’s daily life is required. A noise reduction method based on SCNMF for biological sound measurement was proposed in our previous study. This method may distort the biological sound signal because the correlation between the basis matrices of the signal and noise was not considered. In this paper, we proposed a novel noise reduction system based on OCNMF, in which each vector of the basis matrix imposed the constraint of maximizing the cosine distance. The effectiveness of the proposed method was verified by experimentally comparing the SNR and SDR with the conventional method. The experimental results indicated that the SNR was significantly improved by 1.4 dB on average over the conventional method and the SDR by 2.1 dB on average. The findings proved that the proposed OCNMF-based system is advantageous for biological sound measurement in noisy environments. The proposed method can be applied to systems that assist medical professionals in diagnosis and automatically evaluate a user’s health condition.

To utilize our proposed method for automatic diagnosis, it is necessary to reconstruct the phase of the biological sound signal and improve the speed of the model. In the future, we will extend the model to include phase estimation and attempt to improve its speed based on the sparsity of the matrix. In addition, we will study the optimization of the number of bases to cope with a large number of noise species.

## Figures and Tables

**Table 1:** Specification of frequency filters for vascular sound signal (VSS) and respiratory sound signal (RSS).

Target	Frequency [Hz]	Response	Filter	Order
VSS	75–200	Infinite impulse response	Butterworth	12
RSS	200–2000			

**Table 2:** Characteristics of subjects.

Target	Age [years]	Gender	Disease
A	73	Male	Asthma and chronic obstructive pulmonary disease
B	74	Male	Chronic obstructive pulmonary disease
C	73	Male	Asthma
D	72	Male	Chronic obstructive pulmonary disease
E	72	Female	Asthma
F	74	Male	Asthma
G	77	Male	Chronic obstructive pulmonary disease
H	87	Male	Chronic obstructive pulmonary disease
I	62	Female	Asthma
J	58	Female	Asthma and Chronic obstructive pulmonary disease
K	65	Female	Asthma
L	72	Female	Asthma and chronic bronchitis
M	72	Male	Chronic obstructive pulmonary disease
N	63	Male	Asthma and chronic bronchitis
O	56	Male	Asthma and chronic bronchitis
P	81	Female	Asthma
Q	57	Female	Chronic obstructive pulmonary disease
R	24	Male	No disease
S	24	Male	No disease
T	24	Male	No disease
U	22	Male	No disease

**Table 3:** Predetermined parameters in short-time Fourier transform (STFT), harmonic percussion sound separation (HPSS), CNMF, SCNMF, and OCNMF.

Sampling frequency	44.1 kHz
Bit depth	16 bits
Window function in STFT	Hann window
Window length in STFT	1024 points
Shift length in STFT	512 points
Input SNR	0 dB
Parameters in HPSS	$m_{t}$ = 23
Parameters in CNMF	*R* = 30, *K* = 10, $i_{\mathrm{CNMF}}$ = 200, *β* = 2
Parameters in SCNMF	*J* = 15, *K* = 10, $i_{\mathrm{SCNMF}}$ = 200, *β* = 2
Parameters in OCNMF	*J* = 15, *K* = 10, $i_{\mathrm{OCNMF}}$ = 200, *β* = 2, *μ* = 1.0 × 10^6^

## Appendix A

Table A1 shows the SNR and SDR for each subject in the case of sine wave noise and Table A2 shows the SNR and SDR for each subject in the case of male voice noise.

**Table A1:** SNR and SDR for each subject in the case of sine wave noise.

Subject	SNR [dB]		SDR [dB]	
	SCNMF	OCNMF	SCNMF	OCNMF
A	20.5	20.5	15.9	18.1
B	23.4	24.3	14.8	15.0
C	22.7	24.2	11.3	16.7
D	21.1	22.8	12.4	13.2
E	22.3	25.7	13.2	18.7
F	21.8	21.6	11.5	11.9
G	20.9	22.3	11.2	12.3
H	21.2	22.4	15.7	17.2
I	21.1	24.1	11.2	14.2
J	22.6	23.2	14.1	16.7
K	22.0	24.9	12.1	14.8
L	21.4	22.3	11.5	13.9
M	23.2	24.1	12.3	12.6
N	20.1	21.2	14.2	17.1
O	20.3	21.5	14.8	15.7
P	20.6	22.4	12.0	13.4
Q	21.2	24.3	11.9	14.8
R	21.4	22.6	13.1	16.2
S	21.2	22.9	14.1	17.1
T	20.7	22.1	12.6	15.3
U	21.3	21.4	13.2	14.2

**Table A2:** SNR and SDR for each subject in the case of male voice noise.

Subject	SNR [dB]		SDR [dB]	
	SCNMF	OCNMF	SCNMF	OCNMF
A	19.6	20.8	13.8	16.0
B	21.7	22.4	14.1	13.3
C	21.5	23.5	9.0	16.1
D	18.9	23.7	10.2	11.6
E	20.9	22.9	13.9	18.5
F	20.4	20.9	9.1	12.4
G	21.6	22.4	10.2	10.1
H	21.2	23.5	15.1	17.7
I	21.3	22.8	10.6	14.0
J	22.5	22.5	12.7	14.7
K	22.3	23.4	11.2	13.4
L	22.5	24.1	11.7	12.4
M	22.3	21.0	10.4	12.6
N	21.2	20.8	13.1	14.7
O	18.6	20.7	13.5	15.8
P	19.4	22.9	11.0	12.0
Q	20.6	21.5	12.0	13.0
R	20.1	22.0	13.2	16.9
S	19.8	22.3	14.3	17.1
T	22.0	23.3	11.5	12.8
U	20.6	20.4	13.8	13.1
